# Reducing postprandial glucose in dietary intervention studies and the magnitude of the effect on diabetes-related risk factors: a systematic review and meta-analysis

**DOI:** 10.1007/s00394-020-02240-1

**Published:** 2020-04-10

**Authors:** Carolien Ruijgrok, Ellen E. Blaak, Léonie Egli, Pierre Dussort, Sophie Vinoy, Simone P. Rauh, Joline W. Beulens, M. Denise Robertson, Marjan Alssema

**Affiliations:** 1 Department of Epidemiology & Biostatistics, Amsterdam UMC – location VUmc, Amsterdam Public Health Research Institute, Amsterdam, The Netherlands; 2 Department of Human Biology, NUTRIM, School of Nutrition and Translational Research in Metabolism, Maastricht University, Maastricht, The Netherlands; 3 Nestlé Institute of Health Sciences, Nestlé Research, Lausanne, Switzerland; 4 ILSI Europe a.I.S.B.L., Avenue E. Mounier 83, Box 6, 1200 Brussels, Belgium; 5 Nutrition Department, Mondelez International R&D, Saclay, France; 6 Julius Center for Health Sciences and Primary Care, University Medical Center Utrecht, Utrecht, The Netherlands; 7 Department of Nutritional Sciences, University of Surrey, Guildford, UK; 8 Unilever Research and Development, Vlaardingen, The Netherlands

**Keywords:** Glucose, Insulin, Glycemic index, Glycemic load, HbA1c

## Abstract

**Purpose:**

Reducing postprandial hyperglycemia has beneficial effects on diabetes-related risk factors, but the magnitude of the reduction needed to achieve such an effect is unknown. The purpose of the study was to quantify the relationship of acute glucose and insulin postprandial responses with longer-term effects on diabetes-related risk factors by performing a systematic review and meta-analysis of dietary intervention studies.

**Methods:**

We systematically searched EMBASE and MEDLINE. Dietary intervention studies among any human population aiming to reduce postprandial glycemia, with actual measures of postprandial glucose (PPG) and/or insulin (PPI) as acute exposures (incremental area under the curve, iAUC) as well as markers of glucose metabolism (fasting glucose, HbA1c) and insulin sensitivity (fasting insulin, HOMA-IR) after at least 4 weeks of diet intervention as outcomes were included. Meta-analyses were performed for the effects on acute exposures and on diabetes-related risk factors. The relationship between changes in acute exposures and changes in risk factor outcomes was estimated by meta-regression analyses.

**Results:**

Out of the 13,004 screened papers, 13 papers with 14 comparisons were included in the quantitative analysis. The dietary interventions acutely reduced mean PPG [mean difference (MD), − 0.27 mmol/l; 95% CI − 0.41 to − 0.14], but not mean PPI (MD − 7.47 pmol/l; 95% CI − 16.79 to 1.86). There were no significant overall effects on fasting glucose and insulin. HbA1c was reduced by − 0.20% (95% CI − 0.35 to − 0.05). Changes in acute PPG were significantly associated with changes in fasting plasma glucose (FPG) [per 10% change in PPG: *β* = 0.085 (95% CI 0.003, 0.167), *k* = 14], but not with fasting insulin [*β* = 1.20 (95% CI − 0.32, 2.71), *k* = 12]. Changes in acute PPI were not associated with changes in FPG [per 10% change in PPI: *β* = − 0.017 (95% CI − 0.056, 0.022), *k* = 11].

**Conclusions:**

Only a limited number of postprandial glucose-lowering dietary intervention studies measured acute postprandial exposures to PPG/PPI during the interventions. In this small heterogeneous set of studies, an association was found between the magnitude of the acute postprandial responses and the change in fasting glucose, but no other outcomes. More studies are needed to quantify the relationship between acute postprandial changes and long-term effects on risk factors.

**Electronic supplementary material:**

The online version of this article (10.1007/s00394-020-02240-1) contains supplementary material, which is available to authorized users.

## Introduction

Obesity and type 2 diabetes (T2D) are major global concerns. Recent estimates of T2D expect dramatic increases by 2035 to reach 471 million of cases globally [1]. Postprandial hyperglycemia, as well as the related phenomena of hyperinsulinemia and hyperlipemia, has been implicated in the etiology of chronic metabolic diseases such as T2D [1]. Moreover, elevated fasting and postprandial glucose levels are consistently associated with an increased risk of cardiovascular events, even in the non-diabetic range [3]. To prevent diabetes, an integrated approach is required which includes both dietary modification and regular physical activity [4–6]. Indeed, in non-diabetic hyperglycemia, lifestyle treatment or medication to improve glycemic control was associated with a reduced risk of future diabetes [7].

A number of papers have hypothesized the value of consuming low glycemic index foods to decrease the overall glycemic response of the diet for long-term benefit. Meta-analyses of the effect of low glycemic index (GI) diets indeed demonstrated beneficial effects on body weight in people with obesity and prevention of T2D and cardiovascular diseases [8–10].

However, the magnitude of the reduction of postprandial glycemic response using dietary interventions such as low GI foods or meals, compared to high GI interventions in relation to longer-term established diabetes-related risk factors has not been quantified. At the moment, the majority of dietary studies investigate individual foods and their ability to reduce glucose levels over a period of a single meal only. It is therefore important to understand the relevance of these single meal studies by investigating the quantitative reductions in PPG/PPI needed acutely to induce relevant changes on established longer-term risk factors chronically, and disease prevention ultimately. Therefore, the aim of this work was to quantify the relationship between acute glucose and insulin postprandial responses and their effects on diabetes-related risk factors over time by performing a systematic review and meta-analysis of controlled postprandial glucose-lowering dietary intervention studies.

## Methods

### Data source and searches

The bibliographic databases Elsevier Medical Database (EMBASE) and the US National Library of Medicine database (MEDLINE via the PubMed portal) were systematically searched for relevant papers until September 13, 2019. Relevant papers that were identified while developing the search string or based on authors’ own files were manually included when needed. Search terms were defined by the research question, including terms for GI/glycemic load (GL) dietary interventions, postprandial responses, and study design. Indexed terms were used from MeSH in PubMed and from EMtree in EMBASE. Free-text terms were used in both databases as well. The full search strategies for both databases can be found in Supplementary File 1. The protocol and search strategies used were registered at PROSPERO prior to the study being executed (CRD42018093153).

### Study selection

Titles and abstracts were screened in duplicate, independently by pairs of reviewers (MA, JWB, JMD, LE, CR, FS, SV, MDR) and differences were resolved by consensus. Full-text papers were screened independently by two reviewers (MA, JWB, LE, MDR, CR) for eligibility. Studies were included if they: (1) studied any human population, including healthy individuals and individuals with prediabetes, type 1 and type 2 diabetes mellitus; (2) involved any dietary intervention that aimed at reducing GI, GL, or postprandial glucose responses; (3) reported measures of postprandial glucose (PPG) or postprandial insulin (PPI) as acute exposures to study diets; (4) reported measures of glycemic control and/or insulin sensitivity over time as outcomes. Studies were excluded if they: (1) had a study duration < 4 weeks; (2) were not written in the English language; (3) had no control group; (4) had co-interventions; (5) had changes in glucose-lowering medication use during study; (6) had no accessible full text. If eligible full-text papers did not report acute PPG and PPI response data, papers were checked for references to related papers that had previously published this data. Multiple arms of the same study were included when these arms were independent (had different control groups) [11].

### Data extraction

Data extraction of the included studies was performed by one reviewer (CR) and was appraised (for a random subsample) by a second reviewer (MA). Information on study design, population, intervention diet, acute PPG and PPI exposures (levels per time point, AUC, incremental AUC (iAUC) and outcome measures (markers of glycemic control and insulin sensitivity) were extracted. In case of missing data on exposures and outcomes, the authors were contacted to provide the required information. If the authors did not respond and relevant information was available in figures (i.e., bars for AUC, and responses per time point from graphs), data were extracted from figures using the Microsoft Excel add-in tool TM Image-to-data (tushar-mehta.com).

### Quality assessment

Two reviewers (CR and MA) independently assessed the methodological quality of full-text papers using the Cochrane Risk of Bias Tool [12]. Differences in scores were resolved by consensus. Potential risk of bias was assessed by scoring seven different items (random sequence generation, allocation concealment, blinding of participants and personnel, blinding of outcome assessment, incomplete outcome data, selective outcome reporting, other sources of bias) with low, high or unclear risk of bias and is presented in Supplementary Figure 1.

### Data synthesis and analysis

Outcome data were extracted if reported for at least five comparisons. The exposure and outcome measures glucose and insulin, with variance measure were transformed into SI units [mmol/l for glucose (= 0.0555 × mg/dl) and pmol/l for insulin (= 6 × microU/ml)].

In case postprandial responses were reported as data per time point (in table or as a figure), iAUCs were calculated by the trapezoidal method as net iAUC [13]. Relative changes in exposures PPG and PPI were calculated as:

$$\frac{{{\text{iAUC}}_{{{\text{intervention}}}} {\text{~}} - {\text{iAUC}}_{{{\text{control}}}} }}{{{\text{iAUC}}_{{{\text{control}}}} }} \times 100\% .$$

The outcome was a mean difference between intervention and control. Baseline and post-intervention means with standard deviations (SD) or standard error of the mean (SEM) for the intervention and control groups were extracted, transforming SD into SEM (SEM = SD/√*N*, where *N* = subject population). When actual *P* values were reported, these were used to estimate the SEM [11]. In parallel studies, the absolute change in outcomes was calculated by subtracting the change from baseline in the control group from the change from baseline in the intervention group. In crossover studies, the post-intervention measure of the control group was subtracted from the post-intervention measure of the intervention group. The variance of the absolute changes in outcomes was calculated as ($\sqrt {{\text{SE}}_{{{\text{intervention}}}}^{2} + {\text{SE}}_{{{\text{control}}}}^{2} }$) for parallel studies and ($\sqrt {{\text{SE}}_{{{\text{intervention}}\,{\text{end}}}}^{2} + {\text{SE}}_{{{\text{control}}\,{\text{end}}}}^{2} - 2r \times {\text{SE}}_{{{\text{intervention}}\,{\text{end}}}} \times {\text{SE}}_{{{\text{control}}\,{\text{end}}}} }$) for crossover studies, assuming a within-subject correlation coefficient of 0.8.

Random effects meta-regression analyses were conducted (if number of comparisons *k* > 10) to estimate the association between changes in the acute PPG/PPI exposures and changes in longer-term risk factor outcomes. As additional analyses, overall effects on the acute postprandial exposures and on the outcome variables were estimated by meta-analyses and illustrated by forest plots. In these additional analyses, the postprandial exposures were expressed as mean postprandial levels, calculated as iAUC divided by time. The *Q* test (Chi^2^ statistic, *P* < 0.05) was used to evaluate between-study heterogeneity in meta-analysis and the residual heterogeneity in meta-regression analysis. The *I*^2^ statistic was used for quantification of the degree of heterogeneity and is interpretable as the percentage of the total association that may be due to heterogeneity between studies (*I*^2^ > 50% was considered a meaningful level of heterogeneity) in meta-analysis and as the residual heterogeneity in meta-regression analysis after correction for the changes in acute PPG/PPI exposures. The Pearson correlation coefficient between the change in PPG and the change in PPI was calculated. Bubble charts were created to visualize the relationship between the % relative change in PPG/PPI and the change in diabetes-related risk factors. Planned subgroup analyses stratified by normal versus abnormal glucose metabolism (non-diabetic hyperglycemia or diabetes) could not be conducted (because of *k* comparisons ≤ 10 per subgroup). Instead, for each comparison, normal versus abnormal glucose metabolism was marked by color in the bubble charts (abnormal glucose metabolism was defined on a study group level as being either impaired fasting glucose and/or impaired glucose tolerance and/or HbA1c > 5.7 (%) and/or use of glucose-lowering medication).

Meta-analysis was conducted in Review Manager (RevMan version 5.3. Copenhagen): The Nordic Cochrane Centre, The Cochrane Collaboration, 2014). Meta-regression analysis was performed in R version 3.4.2 using the Metafor package.

## Results

**Fig. 1:**
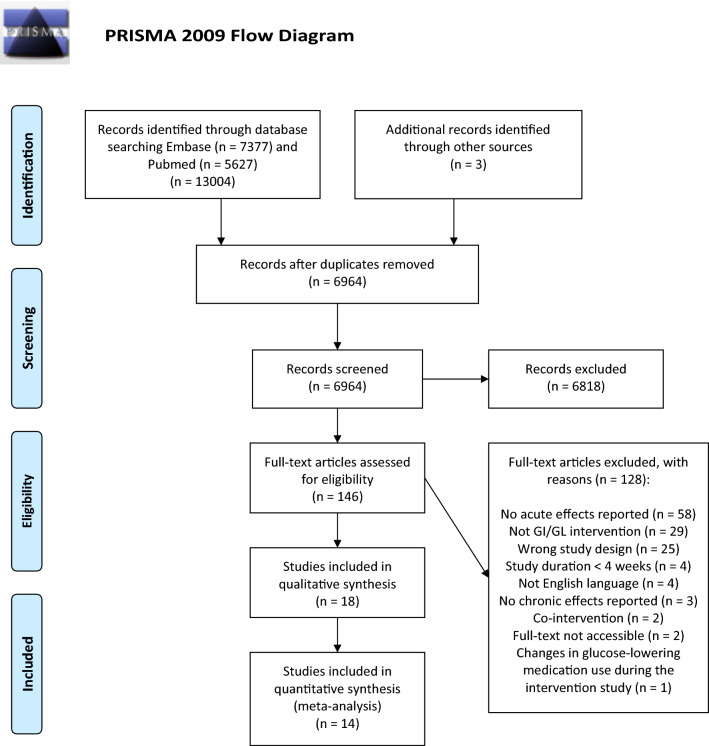
PRISMA flowchart of study inclusion. *PRISMA* Preferred Reporting Items for Systematic Reviews and Meta-Analysis, *GI* glycemic index, *GL* glycemic load

The search retrieved 13,004 papers and an additional 3 potentially relevant papers were found manually and added to the database for screening (Fig. 1). After removal of duplicates, 6964 papers were screened based on titles and abstracts; 146 full-text papers were finally assessed for eligibility. The main reasons for exclusion were: acute effects not reported (58 out of 128 excluded papers), not a PPG-lowering dietary intervention, and not a controlled trial. A total of 17 studies were eligible, of which 13 papers delivered all relevant data needed for quantitative analyses [14–25]. Three studies reported acute and chronic effects of the same dietary intervention in different papers [18, 26, 27] and [22, 28, 29]. One paper [21] reported data from two intervention and two control diets, thereby adding two independent comparisons. The total number of comparisons retrieved from the included set of papers for the quantitative analyses was 14. For PPG, there were 14 comparisons with outcome FPG, 12 with fasting insulin, and 7 with HbA1c. For PPI, there were 11 comparisons with outcome FPG, 10 for fasting insulin, and 4 for HbA1c.

Table 1 summarizes the characteristics of the studies included in the quantitative analyses. Two out of 14 comparisons aimed to reduce postprandial glucose via mulberry leaf extract supplementation [14, 19], while the other comparisons were dietary interventions of whole diet low GI (LGI) versus high GI (HGI) (6 comparisons), low GI breakfast (1 comparison), carbohydrate-reduced high-protein diet (1 comparison), type of rice (2 comparisons) and liquid carbohydrate-modified supplement (2 comparisons). At baseline, five comparisons (four studies) included individuals with normal glucose metabolism and nine comparisons included individuals with abnormal glucose metabolism. The study duration ranged from 4 weeks to 3 months. The intervention was applied to ≥ 3 main meals in nine comparisons, and to < 3 main meals in five comparisons. The duration of postprandial measurement ranged from 120 to 540 min, with a median and most frequent duration of 180 min.

**Table 1:** Characteristics of the included diet studies

Author, year, country		Characteristics of participants	Study design	Duration	Intervention	Control	Provision of meals/products	Effect measure
Asai et al. (2011), Japan	Acute test	2 F and 8 M subjects with abnormal glucose metabolism (50.0 ± 10.6 years) BMI 24.3 ± 1.7 kg/m^2^	Randomized crossover	120 min	Carbohydrate tolerance test—200 g boiled white rice with 2 g of dry seasoning (311 kcal, 70 g CHO, 4.8 g protein, 1.3 g fat) 15 min after ingestion of a mulberry leaf extract capsule (6 mg DNJ)	Carbohydrate tolerance test with placebo capsule		iAUC glucose, iAUC insulin
	Chronic intervention	22 F and 43 M subjects with abnormal glucose metabolism (53.6 ± 6.4 years) BMI 24.6 ± 2.5 kg/m^2^	Randomized parallel	12 weeks	*Diet*—*applied to three main meals* Mulberry leaf extract (6 mg DNJ) capsules were ingested t.i.d. before meals	*Diet*—*applied to three main meals* Placebo capsules were ingested t.i.d. before meals	The mulberry leaf extract and placebo capsules were provided	FPG, fasting insulin, HbA1c
Bouche et al. (2002), France	Acute test	11 M subjects with normal glucose metabolism (mean 46 ± 9.9 years) BMI 28 ± 3.3 kg/m^2^		240 min	LGI breakfast (38%). The breakfast had the same LGI percent as the diet for the chronic period	HGI breakfast (75%). The breakfast had the same HGI percent as the diet for the chronic period		iAUC glucose, iAUC insulin
	Chronic intervention	Same as above	Randomized crossover	5 weeks	*Whole diet approach* LGI diet: foods with a GI < 45%	*Whole diet approach* HGI diet: foods with a GI > 60%	Special cereals and LGI cookies were provided, otherwise participants were supplied with a list of recommended daily intake of commonly used foods and a substitution list allowing exchanges within food groups	FPG, fasting insulin, HOMA
Giacco et al. (2014), Italy	Acute test	31 F and 23 M subjects with normal glucose metabolism (57.2 ± 8.3 years) BMI 31.8 ± 5.6 kg/m^2^		120 min	Lunch meal resembling the composition of the recommended diet before start of the intervention	Lunch meal resembling the composition of the recommended diet before start of the intervention		iAUC glucose, iAUC insulin
	Chronic intervention	Same as above	Randomized parallel	12 weeks	*Diet*—*applied to three main meals* Whole-grain cereal products diet	*Diet*—*applied to three main meals* Refined cereal products diet	Cereal products represented 60–80% of the daily CHO intake; the remaining 20–40% were provided by fruits and vegetables Test products in both diets were provided	FPG, fasting insulin, HOMA-IR
Kabir et al. (2002), France	Acute test	13 M subjects with abnormal glucose metabolism (59 ± 7.2 years) BMI 28 ± 3.6 kg/m^2^		180 min	LGI breakfast that was the same as during the intervention period	HGI breakfast that was the same as during the intervention period		iAUC glucose, iAUC insulin
	Chronic intervention	Same as above	Randomized crossover	4 weeks	*Diet*—*applied to one main meal* LGI breakfast (GI 40%)	*Diet*—*applied to one main meal* HGI breakfast (GI 64%)	Treatment foods for breakfasts were provided during the study (20% of daily energy requirements). Patients were recommended to consume 55% CHO, 15% protein, 30% fat	FPG, fasting insulin, HbA1c
Kallio et al. (2007) and Kallio et al. (2008), Finland	Acute test	9 F and 10 M subjects with metabolic syndrome BMI 31.9 ± 0.7 kg/m^2^		180 min	The test meal consisted of oat and wheat breads or rye breads, 40 g cucumber, and 3 dl of a no-calorie orange drink	The test meal consisted of rye breads, 40 g cucumber, and 3 dl of a no-calorie orange drink		iAUC glucose, iAUC insulin
	Chronic intervention	23 F and 24 M subjects with abnormal glucose metabolism (55.1 ± 6.4 years) BMI 32.0 ± 2.8 kg/m^2^	Randomized parallel	12 weeks	*Whole diet approach* Oat-wheat potato diet	*Whole diet approach* Rye-pasta diet	Participants replaced their normal breads and baked products with the test breads provided during the study (> 25% daily energy intake). Pasta and powdered mashed potatoes were provided	FPG, QUICKI
Kim et al. (2014), Korea	Acute test	23 F and 15 M subjects with abnormal glucose metabolism (51.6 ± 7.5 years) BMI 25.3 ± 3.2 kg/m^2^		120 min	A high-CHO meal in the morning (76 g of white bread and 24 g strawberry jam, 407 kcal, 80 g CHO, 8 g protein, 9.7 g fat) followed within 15 min by MLAE tablet (407 kcal, 80 g CHO, 8 g protein, 9.7 g fat)	A high-CHO meal in the morning (76 g of white bread and 24 g strawberry jam, 407 kcal, 80 g CHO, 8 g protein, 9.7 g fat) followed within 15 min by placebo tablet		iAUC glucose, iAUC insulin
	Chronic intervention	Same as above	Randomized parallel	4 weeks	*Diets*—*applied to three main meals* Six tablets of standardized MLAE with each meal (18 tablets per day: 5 g MLAE (3.6 mg/g of DNJ))	*Diets*—*applied to three main meals* Six placebo (lactose) tablets with each meal (18 placebo tablets per day)	MLAE tablets or placebo tablets provided	FPG, fasting insulin
Mayr et al. (2016), Germany	Acute test	20 F and 20 M subjects with abnormal glucose metabolism (83.0 ± 5.8 years) BMI 23.9 ± 4.0 kg/m^2^		240 min	200 ml carbohydrate modified oral nutritional supplement	200 ml standard oral nutritional supplement		iAUC glucose,
	Chronic intervention	Same as above	Randomized parallel	12 weeks	*Intervention*—*applied two times daily* − 2 × 200 ml/day, in between regular meals, diabetes-specific carbohydrate modified oral nutritional supplement (ONS)	*Control*—*applied two times daily* Standard oral nutritional supplement (ONS) 2 × 200 ml/day in between regular meals	The study nutritional products (ONS) were provided to the subjects	FPG, fasting insulin, HbA1c, HOMA-index
McMillan-Price et al. (2006), Australia	Acute test	11 F subjects (26.5 ± 14.6 years) BMI 30.0 ± 14.3 kg/m^2^	Randomized crossover	180 min	Mixed meals representative of each diet were fed over 10-h period			iAUC glucose, iAUC insulin
	Chronic intervention	98 F and 31 M subjects with normal glucose metabolism (31.8 ± 8.7 years) BMI 31.2 ± 4.6 kg/m^2^	Randomized parallel	12 weeks	*Diets*—*whole diet approach* High CHO (55% E)/HGI High protein (25% E)/HGI	*Diets*—*whole diet approach* High CHO (55% E)/LGI High protein (25% E)/LGI	All key CHO and protein foods and some pre-prepared meals were provided	FPG, fasting insulin, HOMA-IR
Nakayama et al. (2017) and Terashima et al. (2017), Japan	Acute test	13 F and 17 M subjects with abnormal glucose metabolism (61.1 ± 12.5 years) BMI 26.3 ± 3.9 kg/m^2^		180 min	Breakfast with GBR and side dishes (omelet, hamburger, white fish fillet, or salmon)	Breakfast with WR and side dishes (omelet, hamburger, white fish fillet, or salmon)		iAUC glucose
	Chronic intervention	4 F and 12 M subjects with abnormal glucose metabolism (64.0 ± 8.8 years) BMI 25.7 ± 5.6 kg/m^2^	Randomized crossover	8 weeks	*Diets*—*applied to two main meals* Glutinous brown rice twice daily	*Diets*—*applied to two main meals* White rice twice daily		FPG, HbA1c
Nazare et al. (2010), France	Acute test	19 F and 19 M subjects with normal glucose metabolism (38.3 ± 9.2 years) BMI 27.3 ± 1.5 kg/m^2^		270 min	Breakfast consisting of plain biscuits (LGI) with exactly the same composition as those ingested during the study	Breakfast consisting of flakes (HGI) with exactly the same composition as those ingested during the study		iAUC glucose, iAUC insulin
	Chronic intervention	Same as above	Randomized parallel	5 weeks	*Whole diet approach* LGI (GI < 50%) starch diet	*Whole diet approach* HGI (GI > 70%) starch diet	Cereal breakfast products (extruded cereals for the HGI group and plain biscuits for the LGI group), and black bread for the LGI group were provided. A detailed list was given to the participants indicating the starches they were allowed to eat and the prohibited ones	FPG, fasting insulin, HOMA-IR, QUICKI
Samkani et al. (2018) and Skytte et al. (2019), Denmark	Acute test	14 M and 2 F subjects with type 2 diabetes and treated with metformin only (median age 65 (43–70)) BMI 30 ± 4.4 kg/m^2^	Randomized crossover	450 min	Carbohydrate-reduced high protein (31%E carb, 29%E protein, 40%E fat) breakfast (*t* = 0) and lunch (*t* = 270)	Isoenergetic conventional diabetes (54%E carb, 16%E protein, 30%E fat) breakfast (*t* = 0) and lunch (*t* = 270)		iAUC glucose, iAUC insulin
	Chronic intervention	28 M and F subjects with type 2 diabetes	Randomized crossover	6 weeks	Carbohydrate-reduced high protein diet (30%E carb, 30%E protein, 40%E fat)	Isoenergetic conventional diabetes diet (50%E carb, 17%E protein, 33%E fat)	Full diet (five daily meals) were provided	FPG, fasting insulin, HbA1c
Shimabukuro et al. (2013), Japan	Acute test	6 M subjects with the metabolic syndrome (41 ± 5 years) BMI 28.1 ± 4.3 kg/m^2^		240 min	A meal (450 kcal) including BR of Japonica variety (200 kcal)	A meal (450 kcal) including WR of Japonica variety (200 kcal)		iAUC glucose, iAUC insulin
	Chronic intervention	27 M subjects with abnormal glucose metabolism (Age: unknown) BMI 26.7 ± 3.5 kg/m^2^	Randomized crossover	8 weeks	*Diets*—*applied to one main meal* Brown rice of Japonica variety in a single daily meal	*Diets*—*applied to one main meal* White rice of Japonica variety in a single daily meal	Rice was provided during the study	FPG, fasting insulin, HbA1c, HOMA-IR
Stenvers et al. (2014), the Netherlands	Acute test	10 F and 10 M subjects with abnormal glucose metabolism (60 ± 7 years) BMI 30.7 ± 6.4 kg/m^2^		180 min	Low-glycemic response liquid meal (mean of the first 4 days of the intervention period)	Dutch whole-food breakfast was consumed (mean of the first 4 days of the intervention period)		iAUC glucose
	Chronic intervention	Same as above	Randomized crossover	3 months	*Diets*—*applied to one main meal* Low-glycemic response liquid breakfast (isoenergetic amount of Glucerna SR)	*Diets*—*applied to one main meal* Free-choice breakfast	Participants were provided with sufficient amounts of the low-glycemic breakfast in the preferred taste	FPG, fasting insulin, HbA1c

Abnormal glucose metabolism: impaired fasting glucose and/or impaired glucose tolerance and/or HbA1c > 5.7 (%) and/or use of glucose-lowering medication

*BR* brown rice, *CHO* carbohydrate, *DNJ* deoxynojirimycin, *E* energy, *F* females, *FPG* fasting plasma glucose, *GBR* glutinous brown rice, *GI* glycemic index, *HGI* high glycemic index, *HOMA-IR* Homeostatic Model Assessment for Insulin Resistance, *LGI* low glycemic index, *M* males, *MLAE* mulberry leaf aqueous extract, *ONS* oral nutritional supplement, *PPG* postprandial glucose, *PPI* postprandial insulin, *QUICKI* Quantitative Insulin Sensitivity Check Index, *WR* white rice

The majority of the studies scored a high risk of bias on blinding of participants and personnel (Supplementary Fig. 1). All studies scored a low risk of bias on blinding of outcome assessment and selective reporting. Randomization and allocation concealment scored most frequently an unclear risk of bias.

The acute relative change in iAUC glucose ranged from − 121 to 3.5%, with a median of − 27.1%. The acute relative change in iAUC insulin ranged from − 36.8 to 33.2%, with a median of − 29.2%. The correlation between the change in iAUC glucose and the change in iAUC insulin was 0.69 (*P* = 0.019), see Supplementary Fig. 2.

Overall, the dietary interventions acutely reduced the absolute mean PPG levels (mean difference − 0.27 mmol/l; 95% CI − 0.41 to − 0.14; *P* < 0.0001; Supplementary Fig. 3A), but this effect was not significant for mean PPI level (mean difference − 7.47 pmol/l; 95% CI − 16.79 to 1.86; *P* = 0.12; Supplementary Fig. 3B).

No significant overall chronic effects were found for dietary intervention studies on fasting plasma glucose (mean difference 0.03 mmol/l; 95% CI − 0.27 to 0.33; *P* = 0.83) and fasting insulin (mean difference 3.10 pmol/l; 95% CI − 2.37 to 8.56; *P* = 0.27), but an overall reduction in HbA1c was observed (mean difference − 0.20%; 95% CI − 0.35 to − 0.05; *P* = 0.01) (Supplementary Fig. 4A–C).

**Fig. 2:**
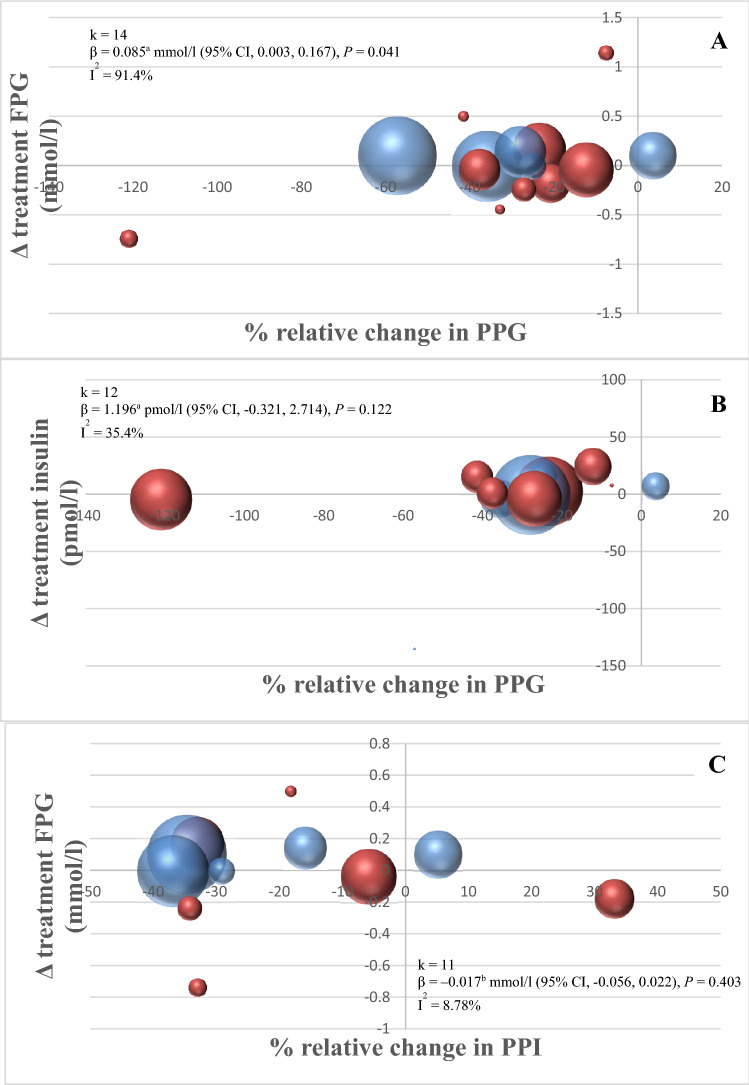
Bubble charts of the relationship between % relative change in PPG and absolute change in **a** FPG and **b** fasting insulin. **c** The relationship between % relative change in PPI and absolute change in FPG. The size of the bubbles indicates the weight of each study (inverse variance); ^a^per 10% change in PPG; ^b^per 10% change in PPI. Random effects meta-regression analyses were conducted (if number of comparisons *k* > 10) to estimate the association between changes in the acute PPG/PPI exposures and changes in longer-term risk factor outcomes. The *I*^2^ statistic was used for quantification of the degree of heterogeneity and is interpretable as the percentage of the total association that may be due to heterogeneity between studies (*I*^2^ > 50% was considered a meaningful level of heterogeneity) in meta-analysis and as the residual heterogeneity in meta-regression analysis after correction for the changes in acute PPG/PPI exposures. Bubble charts were created to visualize the relationship between the % relative change in PPG/PPI and the change in diabetes-related risk factors. For each comparison, normal versus abnormal glucose metabolism was marked by color in the bubble charts (abnormal glucose metabolism was defined on a study group level as being either impaired fasting glucose and/or impaired glucose tolerance and/or HbA1c > 5.7 (%) and/or use of glucose-lowering medication). Meta-regression analysis was performed in R version 3.4.2 using the Metafor package. *FPG* fasting plasma glucose, *PPG* postprandial glucose, *PPI* postprandial insulin

The relationships between % relative acute changes in PPG/PPI and changes in FPG, fasting insulin, and HbA1c are presented in Fig. 2 and Supplementary Fig. 5. Three out of these six relationships had sufficient comparisons/data (*k* > 10) to conduct meta-regression analyses (Fig. 2). Changes in acute PPG responses were associated with changes in FPG (per 10% change in PPG: *β* = 0.085; 95% CI 0.003, 0.167; *k* = 14), but not with fasting insulin (*β* = 1.196; 95% CI − 0.321, 2.714; *k* = 12). Changes in acute PPI responses were not associated with changes in FPG (per 10% change in PPI: *β* = − 0.017; 95% CI − 0.056, 0.022; *k* = 11). By visual inspection, no differences in results were observed between studies with individuals with normal glucose metabolism versus studies with individuals with abnormal glucose metabolism (Fig. 2). Heterogeneity of all meta-analyses and meta-regression results was always below an *I*^2^ of 50% with the exception of the overall effects of the interventions on FPG (96%) and the association between acute PPG response and FPG (91.4%).

## Discussion

This systematic review and meta-analysis of controlled dietary intervention studies aimed to investigate the size of the association between acute PPG and PPI responses and longer-term effects on diabetes-related risk factors. The evidence to examine this association was found to be limited to a set of 13 heterogeneous studies reporting 14 comparisons. An association was found between the size of the reduction in acute PPG exposures to study diets and FPG, but not between PPG and fasting insulin and HbA1c. No associations were found between acute PPI exposures and any of the outcomes.

A strength of this meta-analysis was the systematic approach to identify studies. Moreover, among included studies, the range in both PPG changes (− 121% to + 4%) and PPI changes (− 37% to + 33%) was substantial, which provided enough variation in exposures to potentially identify an association with outcomes. An important limitation was that our systematic review procedure yielded only a small number of studies that actually assessed PPG and PPI exposures to the diets under study. Most studies that aimed to reduce such exposures have designed the study diets based on published GI tables, or assumed effects on PPG, without quantification of actual PPG exposures, and were therefore not eligible for the present review. This perhaps identifies a limitation in the way nutritional research is currently undertaken. The small number of studies reduced study power and precluded analyses of effects on other outcomes (HbA1c). Another limitation of this meta-analysis is that the set of included studies were heterogeneous in study design and the number of studies did not allow for stratification by these sources of heterogeneity. Some major sources of potential heterogeneity were glucose metabolism status and the intensity of the intervention. Indeed, subjects with normal and abnormal glucose metabolism might respond differently to low GI interventions with a greater change in FPG reported previously in subjects with poor glycemic control [9]. The intensity of the intervention varied, as some involved all meals (whole diet approach) and others one meal only, which hampers quantification of PPG exposures during the day. Other potential sources of heterogeneity were study quality, duration of the chronic intervention and compliance to diets.

In our selected set of studies, a significant reduction in HbA1c, but no other longer-term risk factors (fasting glucose and insulin) following PPG-lowering dietary interventions of at least 4 weeks was found. These findings seem to be somewhat at odds with previous GI/GL epidemiologic and some intervention studies. Indeed, several prospective cohort studies have shown an association between GI/GL and the risk of T2D [30–33]. In a meta-analysis of prospective cohort studies, Barclay et al. concluded on an independent effect of GI/GL on the risk of developing T2D [34]. However, due to their observational nature, one cannot exclude the role of confounders (e.g., other dietary factors) in the observed association with T2D.

As reviewed by Blaak et al. results from short-term GI/GL intervention on insulin sensitivity and/or secretion still remain inconclusive [2]. While 11 studies demonstrated a beneficial effect on insulin sensitivity or insulin secretion, 10 papers did not report any difference. Livesey et al. performed a systematic review and meta-analysis of intervention trials on GI and markers of health [9]. They concluded on a favorable effect of consumption of reduced glycemic response diets on reduction of FPG and glycated proteins. However, the effect of low GI interventions seems to vary according to the subjects’ glucose control status. Indeed, the improvement in fasting blood glucose and glycated proteins was reported to be greater in subjects with poor fasting glucose control (> 5 mmol/l). Also, weak evidence suggested a reduction in fasting insulin concentration, only in people who were overweight or obese with fasting insulin concentrations above 100 pmol/l. We did not have sufficient data to tease out the differential effects between individuals with normal versus abnormal glucose metabolism, but the visual inspection did not indicate any differences between studies among individuals with normal versus abnormal glucose metabolism. The discrepancies with Livesey’s meta-analysis may be partially explained by the studies included [9]. Indeed, we only included studies in which the effect on the acute reductions of postprandial glycemia was quantified, while this effect was not assessed in most of the 45 publications included in Livesey et al.’s meta-analysis [9]. Despite the lack of overall effect on fasting glucose, the present study revealed a relationship of PPG with fasting plasma glucose. Given the heterogeneity of the studies and the lack of overall effect on fasting glucose, these results should be interpreted with care. On the other hand, our data do provide some support for a relationship between the intensity of the postprandial glucose response and that of the reduction in fasting glucose.

Although there is abundant evidence that elevated blood glucose, concomitantly with elevated insulin concentration, leads to a transitory deleterious metabolic and hormonal state and oxidative stress, involving the liver, the pancreas, skeletal muscles, lipid metabolism interactions as well as incretins and inflammatory parameters, the exact role of PPG and the relevant magnitude of effect in this process remains unknown [2]. However, it has been postulated that glycemic variability may be a much better indicator for related metabolic effects [35]. Indeed, multiple cohort studies have shown that a high glycemic variability is associated with an increased risk of cardiovascular disease in people with T2D independent of mean plasma glucose or HbA1c [36–38].

Daily exposures to glucose can currently be measured relatively non-invasively via continuous glucose monitoring (CGM) systems. In the present dataset, only one of the included studies utilized this system [28]. In an observational study that used CGM, a positive relationship between PPG and HbA1c was found, both in healthy individuals and those with diabetes yyyy [39]. Further application of CGM in (dietary) intervention studies that aim to reduce glycemic exposure would provide better understanding of achieved reductions in overall PPG exposure and variability. This will enable the estimation of relevant PPG reductions as well as setting benchmarks for PPG exposure in future interventions.

In conclusion, only a limited number of postprandial glucose-lowering dietary intervention studies measure the actual reductions in acute PPG/PPI to the intervention, which they then go on to administer chronically. In this small heterogeneous set of studies, an association was found between the magnitude of the acute postprandial responses and the change in fasting glucose but no other outcomes. To enable setting quantitative benchmarks for PPG/PPI reductions, future dietary intervention studies should consider measuring PPG/PPI exposure to study diets before embarking on a long-term dietary intervention. Similarly, investigators should be encouraged to move beyond the single acute meal study and to follow these up with a chronic intervention, to establish the true effects on metabolic risk.

## Electronic supplementary material

Supplementary file2 (DOCX 795 kb)

## Abbreviations

BR: Brown rice
CGM: Continuous glucose monitoring
CHO: Carbohydrate
DNJ: Deoxynojirimycin
E: Energy
F: Females
FPG: Fasting plasma glucose
GBR: Glutinous brown rice
GI: Glycemic index
GL: Glycemic load
HGI: High glycemic index
HOMA-IR: Homeostatic Model Assessment for Insulin Resistance
iAUC: Incremental area under the curve
LGI: Low glycemic index
M: Males
MD: Mean difference
MLAE: Mulberry leaf aqueous extract
ONS: Oral nutritional supplement
PPG: Postprandial glucose
PPI: Postprandial insulin
PRISMA: Preferred Reporting Items for Systematic Reviews and Meta-Analysis
QUICKI: Quantitative Insulin Sensitivity Check Index
SD: Standard deviation
SEM: Standard error of the mean
T2D: Type 2 diabetes
WR: White rice
