# Supplementary material for: Reducing postprandial glucose in dietary intervention studies and the magnitude of the effect on diabetes-related risk factors: a systematic review and meta-analysis
Source: Eur J Nutr. 2020 Apr 10;60(1):259–73. doi: 10.1007/s00394-020-02240-1 (PMC7867534; doi:10.1007/s00394-020-02240-1)
Supplement: Supplementary file 1 — Supplementary file2 (DOCX 795 kb) [file 394_2020_2240_MOESM1_ESM.docx]

**Supplementary File 1.**

Search strategy

MEDLINE/PubMed

| ((("Glycemic Index"[Mesh] OR "Glycemic Load"[Mesh] OR glycaemic[tiab] OR glycemic[tiab]) |
| --- |
| AND ("Area Under Curve"[Mesh] OR "Postprandial Period"[Mesh] OR area under curve*[tiab] OR area under the curve[tiab] OR AUC[tiab] OR meal test*[tiab] OR postprandial[tiab] OR post-prandial[tiab] OR post meal[tiab] OR post-breakfast[tiab] OR post-lunch[tiab] OR daytime[tiab] OR continuous glucose monitoring[tiab] OR interstitial glucose[tiab] OR glucose profile[tiab] OR insulin profile[tiab]) |
| AND (intervention*[tiab] OR randomized controlled trial[pt] OR controlled clinical trial[pt] OR randomized controlled trials[mh] OR random allocation[mh] OR double-blind method[mh] OR single-blind method[mh] OR clinical trial[pt] OR clinical trials[mh] OR "clinical trial"[tw] OR ((singl*[tw] OR doubl*[tw] OR trebl*[tw] OR tripl*[tw]) AND (mask*[tw] OR blind*[tw])) OR "latin square"[tw] OR placebos[mh] OR placebo*[tw] OR random*[tw] OR research design[mh:noexp] OR comparative study[pt] OR evaluation studies[pt] OR cross-over studies[mh] OR control[tw] OR controll*[tw] OR prospectiv*[tw] OR volunteer*[tw])) |
| NOT (animals[mh] NOT humans[mh])) NOT (animal*[tiab] OR mice[tiab] OR mouse[tiab] OR rats[tiab] OR rat[tiab] OR dog[tiab] OR pig[tiab] OR dogs[tiab] OR animal*[tiab] OR pigs[tiab]) |

EMBASE

| (('glycemic index'/exp OR 'glycemic load'/exp OR glycaemic:ab,ti OR glycemic:ab,ti) |
| --- |
| AND ('area under the curve'/exp OR 'postprandial state'/exp OR ‘area under curve*’:ab,ti OR ‘area under the curve*’:ab,ti OR AUC:ab,ti OR ‘meal test*’:ab,ti OR postprandial:ab,ti OR ‘post-prandial’:ab,ti OR ‘post meal’:ab,ti OR ‘post-breakfast’:ab,ti OR ‘post-lunch’:ab,ti OR daytime:ab,ti OR ‘continuous glucose monitoring’:ab,ti OR ‘interstitial glucose’:ab,ti OR ‘glucose profile’:ab,ti OR ‘insulin profile’:ab,ti) |
| AND ('clinical trial'/exp OR 'triple blind procedure'/exp OR 'double blind procedure'/exp OR 'single blind procedure'/exp OR 'randomization'/exp OR 'placebo'/exp OR 'methodology'/de OR 'comparative study'/de OR 'evaluation study'/de OR 'crossover procedure'/exp OR 'clinical trial':ab,ti OR (singl*:ab,ti OR doubl*:ab,ti OR trebl*:ab,ti OR tripl*:ab,ti)) AND ((mask*:ab,ti OR blind*:ab,ti) OR 'latin square':ab,ti OR placebo*:ab,ti OR random*:ab,ti OR control:ab,ti OR controll*:ab,ti OR prospectiv*:ab,ti OR volunteer*:ab,ti)) |
| NOT (animal*:ab,ti OR mice:ab,ti OR mouse:ab,ti OR rats:ab,ti OR rat:ab,ti OR dog:ab,ti OR pig:ab,ti OR dogs:ab,ti OR animal*:ab,ti OR pigs:ab,ti) |


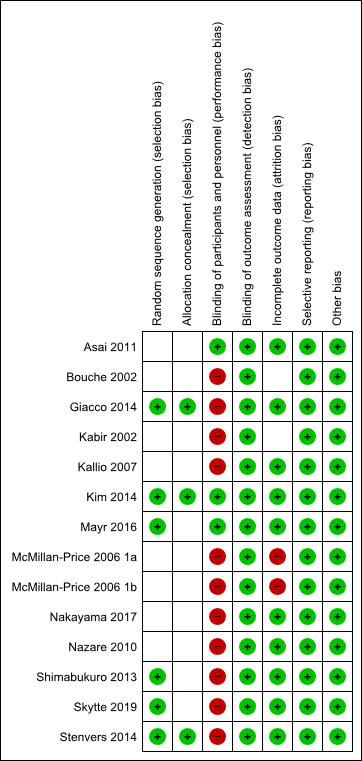


**Supplementary Figure 1.** Risk of bias summary: review authors' judgements about each risk of bias item for each included study. Red dot, high risk of bias. Green dot, low risk of bias.

**Supplementary Figure 2.** Correlation plot. Pearson’s *r* = 0.55, *P* = 0.10

A

**
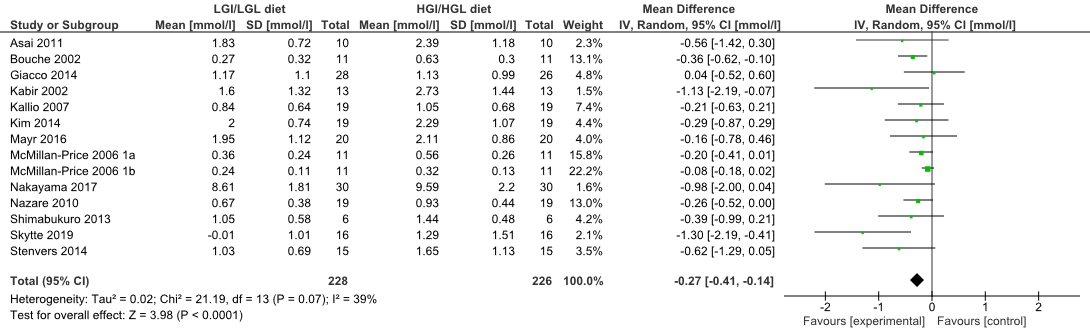
**

B

**
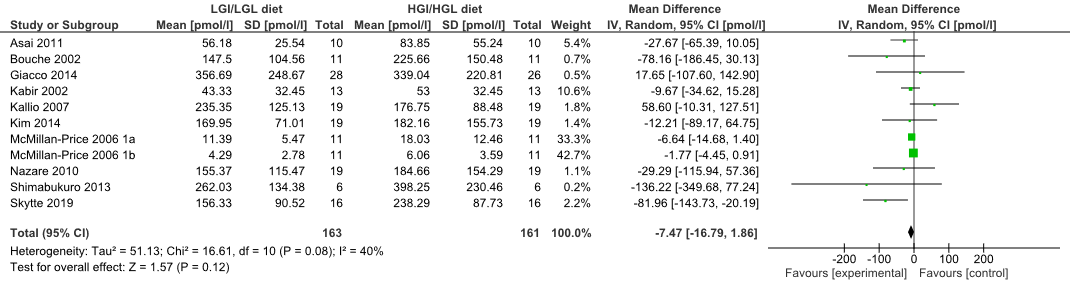
**

**Supplementary Figure 3.** Forest plots of meta-analysis of PPG-lowering dietary intervention on acute (A) mean PPG and (B) mean PPI.

A


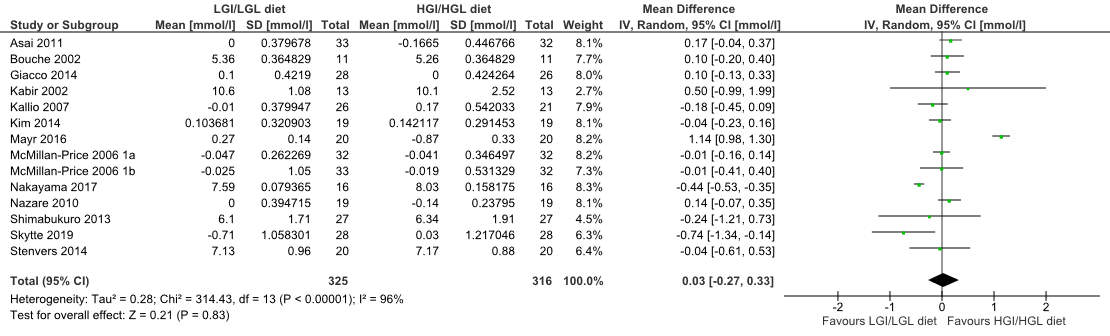


B


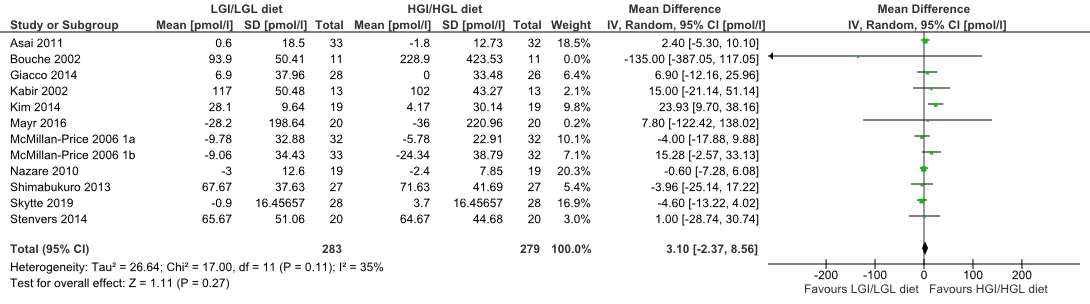


C


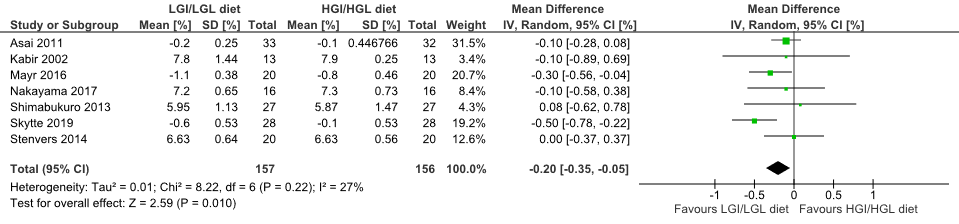


**Supplementary Figure 4.** Forest plots of meta-analysis of PPG-lowering dietary interventions and (A) fasting plasma glucose; (B) fasting insulin; (C) HbA_1c_

**A**

**B**

**C**


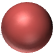

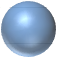


Normal glucose metabolism

Abnormal glucose metabolism

Bubble size: Weight of study (inverse variance)

**Supplementary Figure 5.** Bubble charts of the relationship between % relative change in PPG and absolute change in (a) HbA_1c_ and the relationship between % relative change in PPI and absolute change in (b) insulin (c) HbA_1c_.
